# Author Correction: Hierarchically-structured metalloprotein composite coatings biofabricated from co-existing condensed liquid phases

**DOI:** 10.1038/s41467-020-15462-y

**Published:** 2020-03-31

**Authors:** Franziska Jehle, Elena Macías-Sánchez, Sanja Sviben, Peter Fratzl, Luca Bertinetti, Matthew J. Harrington

**Affiliations:** 1 Department of Biomaterials, Max Planck Institute of Colloids and Interfaces, 14424 Potsdam, Germany; 2 Department of Chemistry, McGill University, 801 Sherbrooke Street West, Montréal, QC H3A 0B8 Canada

**Keywords:** Biopolymers in vivo, Biomaterials, Bioinspired materials

Correction to: *Nature Communications* 10.1038/s41467-020-14709-y, published online 13 February 2020.

The original version of this article omitted from the author list the third author Sanja Sviben, who is from the “Department of Biomaterials, Max Planck Institute of Colloids and Interfaces, 14424 Potsdam, Germany”. Consequently, the new author list and affiliations are as follows:

“Franziska Jehle^1^, Elena Macías-Sánchez^1^, Sanja Sviben^1^, Peter Fratzl^1^, Luca Bertinetti^1^*, Matthew J. Harrington^1,2^*

^1^Department of Biomaterials, Max Planck Institute of Colloids and Interfaces, 14424 Potsdam, Germany. ^2^Department of Chemistry, McGill University, 801 Sherbrooke Street West, Montréal, QC H3A 0B8, Canada. email: luca.bertinetti@mpikg.mpg.de; matt.harrington@mcgill.ca”.

The Author contributions statement was changed to the following to add S.S.: “M.J.H., L.B. and P.F. devised and oversaw the study. L.B., F.J., S.S. and E.M.-S. performed experiments and processed data. M.J.H. and F.J. wrote the paper. All authors contributed to editing the paper”.

This has been corrected in both the PDF and HTML versions of the article.

